# Push–pull–block framework for lycopene biosynthesis: a comprehensive review of metabolic engineering strategies in *Saccharomyces cerevisiae*

**DOI:** 10.3389/fbioe.2026.1887860

**Published:** 2026-08-27

**Authors:** Farah D. Garaad, Hulya Karaca

**Affiliations:** 1 Department of Biology, Eskisehir Technical University, Eskisehir, Türkiye; 2 School of Pure and Applied Sciences, Garissa University, Garissa, Kenya; 3 Department of P. Microbiology, Faculty of Pharmacy, Anadolu University, Eskisehir, Türkiye

**Keywords:** carotenoids, lycopene, metabolic engineering, *Saccharomyces cerevisiae*, synthetic biology

## Abstract

Lycopene is a high-value carotenoid widely utilized in food, pharmaceutical, nutraceutical, and cosmetic industries because of its strong antioxidant properties and associated health benefits. Among microbial hosts, *Saccharomyces cerevisiae* has emerged as a promising platform for sustainable lycopene biosynthesis due to its Generally Recognized as Safe status, well-characterized metabolism, and compatibility with industrial-scale fermentation processes. However, efficient lycopene production in yeast remains constrained by limited precursor availability, metabolic flux imbalance, competing pathways, redox stress, and intracellular toxicity associated with carotenoid accumulation. This review comprehensively summarizes current metabolic engineering strategies for enhancing lycopene biosynthesis in *S. cerevisiae* through an integrated “push–pull–block” framework. Push strategies focus on increasing precursor supply, acetyl-CoA availability, mevalonate pathway flux, and NADPH regeneration. Pull strategies emphasize pathway optimization through promoter engineering, gene dosage tuning, enzyme scaffolding, and heterologous carotenoid pathway enhancement to improve carbon flux toward lycopene biosynthesis. Block strategies target the suppression of competing sterol pathways, relief of feedback inhibition, and redirection of metabolic resources toward carotenoid accumulation. In addition, emerging approaches involving stress mitigation, lipid droplet engineering, adaptive laboratory evolution, dynamic regulation, controlled fermentation, and multi-omics-guided systems metabolic engineering are discussed as next-generation solutions for improving industrial lycopene production. Collectively, this review provides a systems-level perspective on current advances and future opportunities in engineering *S. cerevisiae* as an efficient microbial cell factory for sustainable lycopene biosynthesis.

## 1 Introduction

Metabolic engineering encompasses the systematic modification of cellular networks through genetic and regulatory interventions to enhance, redirect, or enable the biosynthesis of target compounds for industrial applications. These strategies are typically implemented through the overexpression of rate-limiting enzymes, elimination of competing pathways, and introduction of heterologous biosynthetic modules, collectively enabling the efficient production of both native and non-native metabolites (Bailey, 1991; Li et al., 2018). Within this context, microbial cell factories have emerged as versatile and scalable platforms for sustainable bioproduction.

Among microbial hosts, *Saccharomyces cerevisiae* and *Escherichia coli* are the most extensively utilized due to their rapid growth, well-characterized genetics, and cost-effective cultivation. However, *S. cerevisiae* offers distinct advantages, including its Generally Recognized as Safe (GRAS) status, robustness under industrial conditions, and its eukaryotic cellular machinery, which facilitates proper protein folding and post-translational modifications (Kricka et al., 2015; Krivoruchko and Nielsen, 2015). Importantly, its native mevalonate (MVA) pathway provides a favorable metabolic background for the biosynthesis of isoprenoid-derived compounds, positioning *S. cerevisiae* as a preferred host for terpenoid production.

Microbial platforms are increasingly exploited for the biosynthesis of high-value natural products, particularly secondary metabolites such as terpenoids, polyketides, and alkaloids, which are often difficult to synthesize chemically due to their structural complexity and stereochemical requirements (Pickens et al., 2011). Despite their industrial and pharmaceutical significance, the production of these compounds remains constrained by pathway complexity, low precursor availability, and intricate regulatory mechanisms. Terpenoids, the largest and most structurally diverse class of natural products, are synthesized *via* the mevalonate (MVA) or 1-deoxy-D-xylulose-5-phosphate (DXP) pathways, depending on the host organism (Li et al., 2018; Vranová et al., 2013). The inherent complexity and tight regulation of these pathways present significant challenges for metabolic engineering efforts.

Among terpenoids, carotenoids such as lycopene have attracted considerable attention due to their potent antioxidant activity, pigmentation properties, and wide-ranging applications in the food, pharmaceutical, cosmetic, and agricultural industries. Lycopene, classified as a class A nutrient by FAO/WHO, represents a key target for sustainable biotechnological production (Liang et al., 2019). Microbial synthesis offers a controllable and scalable alternative to plant extraction, minimizing environmental variability and enabling precise pathway optimization. Furthermore, enzymatic biosynthesis in engineered microorganisms allows regio- and stereospecific transformations that are often difficult to achieve *via* chemical synthesis, thereby enhancing product bioactivity (Li et al., 2020).

However, achieving high-level lycopene production in *S. cerevisiae* remains challenging due to multiple intrinsic metabolic constraints. These include limited precursor availability (Huang et al., 2024; Li et al., 2019; Su et al., 2022; Zhou et al., 2023), competition with endogenous sterol biosynthesis for farnesyl pyrophosphate (FPP) (Xie et al., 2015; Zhou et al., 2023), redox imbalance (Hong et al., 2019; Huang et al., 2024), metabolic burden associated with heterologous pathway expression (Su et al., 2020; Xie et al., 2015), and the cytotoxic effects of accumulated intermediates and final products (Hong et al., 2019; Huang et al., 2024; Ma et al., 2019; Su et al., 2022). These interconnected limitations necessitate integrated and system-level engineering strategies rather than isolated genetic modifications. To address these challenges, metabolic engineering approaches are increasingly conceptualized within structured frameworks that enable systematic intervention. In this review, we adopt the push–pull–block framework to categorize and critically evaluate strategies for enhancing lycopene biosynthesis in *S. cerevisiae*. This framework encompasses (i) “push” strategies that increase precursor supply, (ii) “pull” strategies that enhance pathway flux toward the desired product, and (iii) “block” strategies that eliminate competing pathways and reduce carbon loss. Despite the extensive body of work in this field, a unified and systematic organization of these strategies remains limited, highlighting the need for a comprehensive and integrative perspective.

Beyond these three core strategies, robust and industrially relevant lycopene production depends on a set of complementary, system-level approaches that do not constitute a separate category but rather reinforce and enable the push, pull, and block components. Cofactor engineering—balancing NADPH, acetyl-CoA, and ATP supply—underpins push strategies, since precursor synthesis through the mevalonate pathway cannot proceed without adequate redox and energy provision. Dynamic pathway regulation reinforces pull by temporally controlling expression so that isoprenoid precursors such as IPP and DMAPP are efficiently channeled toward lycopene rather than accumulating to cytotoxic levels. Stress tolerance optimization sustains stronger pull, allowing cells to withstand the membrane perturbation and oxidative stress imposed by high-level lycopene accumulation, while secretion and intracellular storage strategies (e.g., lipid droplet expansion and membrane engineering) relieve product-associated toxicity and expand the storage capacity that would otherwise limit pull. Finally, adaptive laboratory evolution acts across all three components simultaneously, uncovering non-intuitive, genome-wide optima that rational push–pull–block design alone cannot reach. Framed this way—with rational PPB interventions at the core and these approaches forming an enabling layer around them—genetic, metabolic, and process-level insights integrate into a holistic and forward-looking strategy for developing robust, high-yield, and industrially scalable lycopene-producing *S. cerevisiae* strains.

## 2 Lycopene sources

Lycopene is a highly lipophilic carotenoid characterized by a C_40_ polyene backbone composed of eight isoprene units, containing eleven conjugated and two unconjugated double bonds. This extended conjugated system underlies its distinctive red coloration and potent antioxidant activity (Fraser and Bramley, 2004; Shafe et al., 2024). However, this same structural feature renders lycopene highly susceptible to oxidative degradation and cis–trans isomerization when exposed to environmental stressors such as heat, light, oxygen, and pH variations, ultimately affecting its stability and bioavailability (Boileau et al., 2002; Shi and Maguer, 2000).

Three primary sources of lycopene for food and industrial applications include: (i) extraction from plants, mainly tomatoes; (ii) microbial fermentation; and (iii) chemical synthesis (Li et al., 2020; Zhou et al., 2023). Currently, the majority of commercial lycopene on the market is produced through extraction from tomatoes and microbial fermentation using the fungus *Blakeslea trispora* (Hong et al., 2019; Li et al., 2020; Xie et al., 2015).

### 2.1 Plant extraction

In biological systems, lycopene undergoes isomerization, with cis-isomers exhibiting greater bioavailability than the all-trans forms predominantly found in plant tissues (Boileau et al., 2002; Fraser and Bramley, 2004). Despite its nutritional and functional importance, the availability of lycopene from natural sources remains limited by both biochemical instability and production constraints.

In nature, lycopene accumulates in plant chromoplasts as crystalline–protein complexes (Harris and Spurr, 1969). Tomatoes represent the primary dietary source; however, other fruits such as autumn olive, rosehip, watermelon, guava, grapefruit, mango, and papaya also contribute significant amounts (Böhm et al., 2003; Fraser and Bramley, 2004; Perkins-Veazie et al., 2005; Rao and Agarwal, 2000). Nevertheless, lycopene distribution within plant tissues is often heterogeneous, and its accumulation is influenced by environmental conditions, cultivation practices, and post-harvest processing (Shi and Maguer, 2000).

Lycopene naturally forms hydrophobic microcrystals inside tomato chromoplasts, with the highest concentrations found bound to the insoluble fibers of the skin, making industrial processing waste the primary feedstock for natural extraction (Shi and Maguer, 2000). To isolate the pigment from the protective cellular matrix, modern processes deploy advanced organic solvent, supercritical fluid, enzymatic, or electroporation technologies (Liang et al., 2019; Shi and Maguer, 2000). Because lycopene’s highly unsaturated structure makes it sensitive to thermal and oxidative degradation, extractions require strict environmental controls, though processing heat can beneficially induce a trans-to-cis isomerization that dramatically improves downstream human bioavailability (Shi and Maguer, 2000). While tomato extraction yields high-quality natural lycopene, it is constrained by seasonal variability, geographic dependency, and substantial biomass waste generation. (Li et al., 2020).

### 2.2 Chemical synthesis

Industrial chemical synthesis of lycopene relies on a three-component assembly strategy to build the symmetric C_40_ carotenoid framework from smaller hydrocarbon building blocks. The process begins by converting pseudoionone into a reactive C_15_-phosphonium salt intermediate to form the outer wings of the molecule. Next, a double Wittig condensation couples two equivalents of this generated C_15_- phosphonium salt with a central 2,7-dimethyl-2,4,6-octatriene-1,8-dial (C10-dialdehyde) core. Finally, because this condensation step inherently yields a mixture of isomers, the crude product is subjected to controlled thermal isomerization to transform the *cis*-lycopene structures into the industrially preferred, thermodynamically stable *all-trans* configuration. During this step, the poorly soluble all-trans isomer sequentially crystallizes out of solution and is effectively removed from the isomerization equilibrium before final purification (Ernst, 2002).

Lycopene production through chemical synthesis is technically feasible, but its commercial application is heavily constrained by structural, regulatory, and environmental challenges because of the toxic intermediates used or generated and the resulting environmental pollution. While it offers distinct advantages including, low cost of raw materials, mild reaction conditions, and high reaction yields, due to its highly complex chemical structure, the chemical synthesis process is extremely complicated and requires demanding multi-step pathways and challenges in achieving the stereo-selectivity of the double bonds resulting in stereoisomeric mixtures (Jing et al., 2021; Li et al., 2019; Reyes et al., 2014). Chemical synthesis, compared to plant extraction, enables high-yield production but is limited by poor stereochemical specificity and concerns related to synthetic residues (Li et al., 2020).

### 2.3 Microbial fermentation

Microbial fermentation has emerged as a promising and increasingly competitive alternative to plant extraction and chemical synthesis, offering scalability, process control, reduced environmental impact, and independence from agricultural cycles. Native carotenogenic organisms such as *B. trispora* and red yeasts (e.g., *Rhodotorula* spp.) have demonstrated significant production potential. *Blakeslea trispora*, a filamentous fungus, remains the primary industrially exploited organism for microbial lycopene and β-carotene production, reported to yield up to 256 mg/L of lycopene and capable of utilizing low-cost substrates such as waste cooking oil (Wang et al., 2019). Similarly, oleaginous yeasts provide intracellular lipid storage compartments that facilitate the accumulation of hydrophobic carotenoids while mitigating product-associated toxicity (Ma et al., 2019).

Beyond *B. trispora*, a growing number of alternative microbial hosts have been engineered for lycopene biosynthesis, with substantial variation in reported titers depending on the host organism, pathway design, and cultivation strategy.

By applying a novel combinatorial workflow that pairs Single Strand Assembly (SSA) with Golden Gate Assembly (GGA), researchers achieved a peak intracellular lycopene content in *E. coli* of 448 mg/g lycopene CDW (cell dry weight). This milestone was accomplished by optimizing the expression of the heterologous *crtEIB* gene cluster from *Erwinia herbicola* within a specialized T7-MEP background expression host. The engineered strain yielded a threefold improvement over its respective reference plasmid baseline while simultaneously doubling the lycopene accumulation capabilities previously reported in the literature (Coussement et al., 2017).

The oleaginous, non-conventional yeast *Yarrowia lipolytica* has emerged as an alternative chassis for lycopene production, capitalizing on its robust native acetyl-CoA flux and lipid-storage capabilities (Luo et al., 2023). developed a multi-level genetic framework to optimize lycopene biosynthesis. Their approach utilized a synchronized, two-part engineering network: the heterologous downstream pathway (*crtE*, *crtB*, and *crtI* from *Lamprocystis purpurea*) was established *via* 26 S ribosomal DNA (rDNA)-mediated multicopy random integration, while upstream mevalonate flux was enhanced through iterative, NHEJ-mediated transformations of AtoB and HMGR. This coordinated engineering enabled the optimized strain to accumulate a peak specific lycopene content of 121 mg/g dry cell weight (DCW) and reach a maximum volumetric titer of 5.1 g/L.

Beyond raw titers, host selection for microbial lycopene production is strongly shaped by food-safety and regulatory considerations. While engineered *E. coli* can reach very high titers, its release of endotoxins (Jing et al., 2021; Li et al., 2019; Shi et al., 2019; Watcharawipas et al., 2021) restricts its acceptability in food and nutraceutical applications; similarly, *B. trispora* fermentation typically requires the addition of cyclase inhibitors to prevent conversion of lycopene to β-carotene, complicating downstream processing (Shi et al., 2019; Watcharawipas et al., 2021). These constraints have reinforced interest in GRAS hosts such as *S. cerevisiae*, which combine regulatory acceptability with genetic tractability. Substrate flexibility further differentiates these hosts: *B. trispora* and oleaginous yeasts can utilize low-cost lipid-rich feedstocks such as waste cooking oil and plant oils, whereas engineered *S. cerevisiae* and *Y. lipolytica* can be tailored to assimilate diverse carbon sources including glucose, ethanol, xylose, and acetate. Table 1 summarizes representative lycopene production levels reported across the principal microbial hosts, illustrating the range of titers and yields achieved and the trade-offs associated with each platform.

**TABLE 1:** Comparison of representative microbial hosts for lycopene production, with reported titers, yields, and key characteristics.

Host organism	Type	Reported production	Key characteristics	Reference
*Blakeslea trispora*	Native fungus	Up to ∼256 mg/L; ∼944 mg/L with oil supplementation	Primary industrial organism; requires cyclase inhibitors; uses low-cost lipid substrates	(Sevgili and Erkmen, 2019; Wang et al., 2019)
*Escherichia coli*	Engineered bacterium	448 mg/g DCW	Highest reported contents; endotoxin limits food use; fast growth	(Coussement et al., 2017)
*Saccharomyces cerevisiae*	Engineered yeast (GRAS)	8.15 g/L	GRAS status; genetically tractable; industrial compatibility	(Zhou et al., 2023)
*Yarrowia lipolytica*	Engineered oleaginous yeast	Up to 5.1 g/L; 121 mg/g DCW (fed-batch)	High acetyl-CoA flux; large lipid-storage capacity for hydrophobic products	(Luo et al., 2020 , 2023)
*Rhodotorula* spp.	Native red yeast	Strain-dependent	Native carotenogenic yeast; intracellular lipid storage	(Li et al., 2020)

Despite these advances, these native carotenogenic systems often lack the flexibility required for precise pathway optimization and high-level production. As a genetically tractable but non-carotenogenic host, *Saccharomyces cerevisiae* has therefore emerged as a highly attractive engineered platform for lycopene biosynthesis. Although *Saccharomyces cerevisiae* does not natively produce lycopene, its endogenous mevalonate pathway provides a robust precursor supply, while its genetic tractability enables fine-tuned metabolic engineering interventions (Ma et al., 2019; Xie et al., 2015). Coupled with its GRAS status, compatibility with industrial-scale fermentation, and ability to grow on low-cost substrates, *S. cerevisiae* represents a leading chassis for sustainable lycopene production (Huang et al., 2026; Shi et al., 2019).

Importantly, the limitations associated with natural and native microbial production systems underscore the need for systematic metabolic engineering strategies. These considerations provide the foundation for the application of structured frameworks, such as the push–pull–block approach, to rationally optimize lycopene biosynthesis in engineered yeast systems.

## 3 Lycopene production in *S. cerevisiae*

The integration of classical strain improvement techniques with modern metabolic engineering has led to the development of sophisticated and robust microbial platforms for lycopene production.

In *Saccharomyces cerevisiae*, metabolic engineering efforts for lycopene synthesis capitalize on the organism’s native mevalonate (MVA) pathway, while incorporating heterologous genes for downstream carotenoid biosynthesis (Ma et al., 2019). The MVA pathway provides the essential isoprenoid precursors—dimethylallyl pyrophosphate (DMAPP) and isopentenyl pyrophosphate (IPP)—which are condensed to form geranyl pyrophosphate (GPP), farnesyl pyrophosphate (FPP), and finally geranylgeranyl pyrophosphate (GGPP). The latter serves as a direct precursor for lycopene synthesis (Xu et al., 2021).

The introduction of genes encoding CrtE, CrtB, and CrtI enables the conversion of FPP to lycopene *via* GGPP and phytoene intermediates. These steps involve sequential condensation and desaturation reactions, requiring tight metabolic regulation (Watcharawipas et al., 2021). Efficient production depends on maintaining balanced pools of metabolic precursors including acetyl-CoA, pyruvate, and glyceraldehyde-3-phosphate (G3P). Strategies such as the overexpression of acetyl-CoA synthetase, enhanced peroxisomal metabolism, and elimination of competing pathways have proven effective in redirecting flux toward lycopene biosynthesis (Hong et al., 2019; Kulagina et al., 2021; Shi et al., 2019).

Additionally, the toxic accumulation of IPP and DMAPP can impair cell growth and product yield. Overexpressing the *idi* gene, which facilitates their interconversion, mitigates this metabolic burden (Eun and Lee, 2024). Collectively, these targeted interventions in central carbon and isoprenoid metabolism illustrate the potential of *S. cerevisiae* as a high-efficiency, genetically tractable host for high level lycopene production (Eun and Lee, 2024; Ma et al., 2019).

We categorized lycopene optimization strategies in *S. cerevisiae* using the push–pull–block framework, which structures efforts into precursor supply, pathway enhancement, and competitive pathway suppression—while also covering supportive strategies like stress adaptation and secretion improvement. Within this framework, push encompasses strategies that increase the upstream supply of carbon precursors and redox cofactors feeding the MVA pathway, from acetyl-CoA generation to the strengthening of mevalonate flux, whereas pull targets the downstream conversion of these precursors into lycopene—optimizing carotenoid enzyme performance, pathway organization, and expression control. Block, in turn, removes competing carbon sinks and regulatory brakes, such as flux diversion toward sterol biosynthesis at the FPP node, that constrain flux at either stage.

## 4 Push–Enhancing precursor and cofactor supply

**FIGURE 1:**
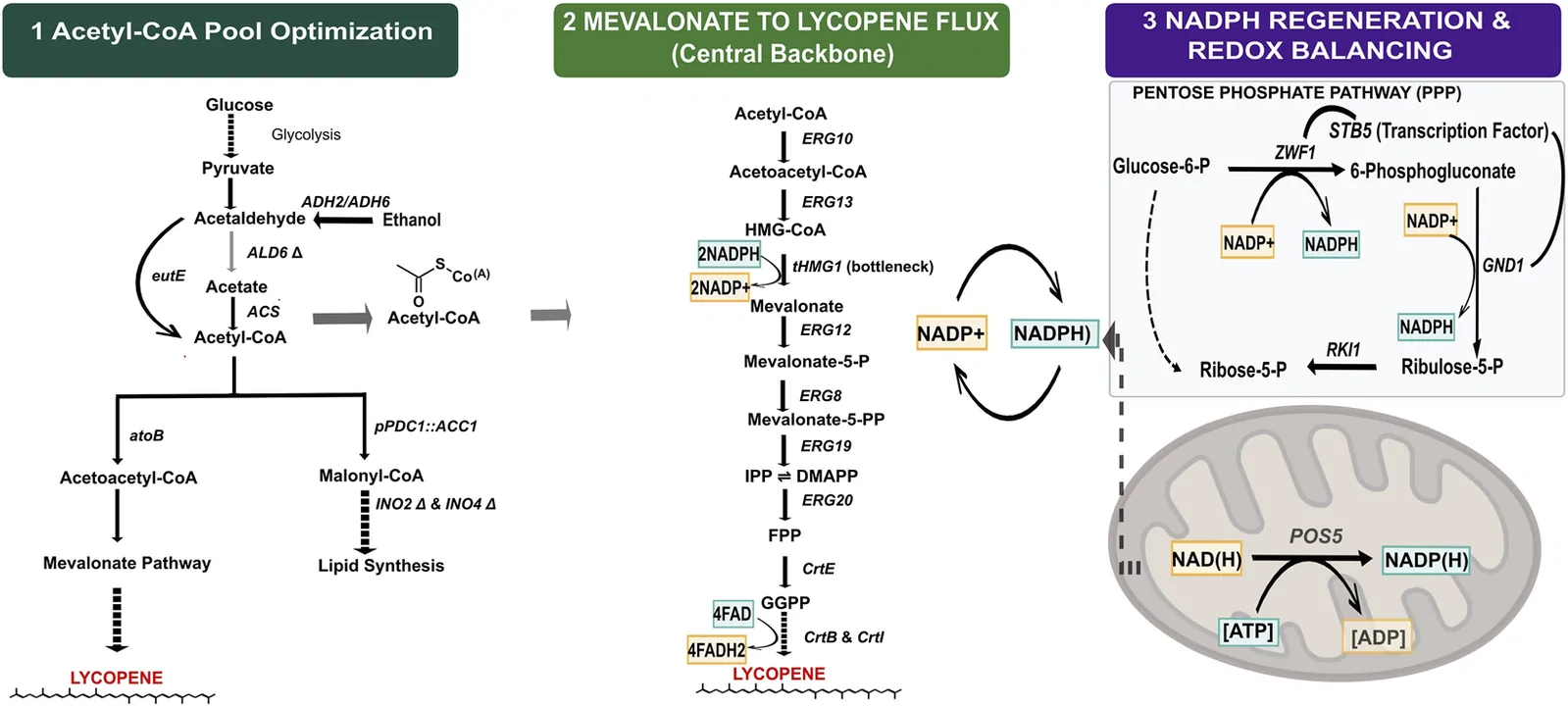
Systematic engineering modules for push strategies to enhance acetyl CoA flux, enhance mevalonate pathway and cofactor balancing. (1) Acetyl-CoA Pool Optimization: Focuses on expanding the cytosolic acetyl-CoA supply to feed the upstream pathways. (2) Mevalonate to Lycopene Flux (Central Backbone): Outlines the downstream synthesis backbone requiring multiple reducing equivalents NADPH and FAD to successfully accumulate lycopene. (3) NADPH Regeneration & Redox Balancing: Targets cofactor supply through the oxidative branch of the Pentose Phosphate Pathway (PPP)—driven by the transcription factor *STB5* alongside Zwf1 and Gnd1 cascades—and ATP-driven mitochondrial conversion *via* the NADH kinase Pos5.

The “push” strategy in metabolic engineering focuses on increasing the intracellular availability of key precursors and cofactors that drive biosynthetic flux toward the target product. In the context of lycopene production in *Saccharomyces cerevisiae*, this primarily involves enhancing cytosolic acetyl-CoA levels, which serve as the fundamental carbon entry point into the mevalonate (MVA) pathway (Figure 1).

### 4.1 Acetyl-CoA pool optimization

Cytosolic acetyl-CoA plays a central role in lycopene biosynthesis, acting as the primary precursor for isoprenoid production *via* the MVA pathway. However, its intracellular availability is tightly regulated and often limited due to compartmentalization and competing metabolic demands. To overcome these constraints, metabolic engineering strategies have been developed to enhance acetyl-CoA availability through three major approaches: increasing precursor supply, improving carbon recycling, and reducing competing consumption.

Enhancing precursor supply has proven to be an effective strategy to boost acetyl-CoA levels. For instance, Su et al. (2022) introduced the bacterial genes *eutE* (acetaldehyde dehydrogenase) and *atoB* (acetyl-CoA acetyltransferase), redirecting carbon flux from pyruvate and achieving a 21-fold increase in mevalonate production. Similarly, deletion of transcriptional regulators *INO2* and *INO4*, which are involved in phospholipid biosynthesis, redirected carbon flux toward the isoprenoid pathway, leading to enhanced lycopene accumulation Su et al. (2024). In addition, disruption of *ALD6*, which prevents acetate formation, further increased cytosolic acetyl-CoA availability and improved lycopene titers Su et al. (2020) (Table 2).

**TABLE 2:** Summary of metabolic engineering strategies for lycopene production in *Saccharomyces cerevisiae*, organized according to the push–pull–block framework, with the key genetic modifications and the corresponding titers and yields reported in each study.

Summary of push strategies for lycopene production in *S. cerevisiae*				
​	Key Genetic Modifications		Titer (mg/L)	Yield	References
Acetyl-CoA Pool Optimization, Mevalonate Pathway Engineering and Cofactor Engineering	*STB5*		120 mg/L	41.8 mg/g CDW	(Hong et al., 2019)
	*tHMG1*, *ALD6*, ACS, *POS5*		2.37 g/L	73.3 mg/g CDW	(Ma et al., 2019)
	*tHMG1*, additional copy of PaCrtE and BtCrtI, *POS5*, *ALD6*, ACS, *ADH2*		3.28 g/L	not reported	(Shi et al., 2019)
	Δ*ALD6*, ΔPHMG1 −HMG1::PCit1−*tHMG1*, ΔPERG9::PPDC1		1.48 g/L	33.1-mg/g CDW	(Su et al., 2020)
	Eut, *atoB*, ΔPACC1::PPDC1		2.61 g/L	68 mg/g CDW	(Su et al., 2022)
	*tHMG1*, ARTP and ALE → (*ADH2*, *ALD4*, and *ACS1*)		8.15 g/L	69.6 mg/g CDW	(Zhou et al., 2023)
	Δ*ALD6*, Δ*INO2* and Δ*INO4*		not reported	11.8 mg/g CDW	(Su et al., 2024)
	Δ*MLS1*, *ERG10*, *ERG12*, *ERG20*, *tHMG1*, *POS5*		343.7 mg/L	47.9 mg/g DCW	(Huang et al., 2024)
Summary of Pull Strategies for Lycopene Production in *S. cerevisiae*					
Carotenoid Pathway (Crt) Engineering	RIAD and RIDD peptide scaffolds (Idi*-*CrtE)		2.3 g/L	not reported	(Kang et al., 2019)
	Fusion Protein Strategy (*crtE–crtB*)		not reported	10.12 mg/g DCW	(Xu et al., 2021)
Enzymes From Different Species	*TmCrtE + PaCrtB + BtCrtI*		1.65 g/L	55.56 mg/g DCW	(Chen et al., 2016)
	*PaCrtE + PagCrtB + BtCrtI*		3.28 g/L	not reported	(Shi et al., 2019)
	*TmCrtE+ PaCrtB + SpCrtI*		120.36 mg/L	20.63 mg/g DCW	(Watcharawipas et al., 2021)
	*PaCrtE + PagCrtB + BtCrtI*		343.7 mg/L	47.9 mg/g DCW	(Huang et al., 2024)
Promoter and Gene Copy Tuning	​		​	​	​
A) Constitutive Promoters	*P*_*TEF1*_ *-*CrtB, P_*FBA1*_ *-*CrtI + P_ADH1_, P_*ALA1*_ *, P*_*FBA1*_ *, P*_*TDH3*_ *, P*_*TYS1*_, and P_*GPM1*_—for upstream precursor genes (*ERG10, ERG13, HMG1, ERG12*, and *ERG8*		115.64 mg/L	12.58 mg/g DCW	(Li et al., 2019)
B) Hybrid Promoter Systems	*P*_*TDH3*_ *-crtB* (constitutive) + *P*_*GAL1*_ *-crtI* + *P*_*GAL10*_ *-crtE*		not reported	10.12 mg/g DCW	(Xu et al., 2021)
C) inducible Promoters	*P*_*GAL1/10*_ *-*CrtE + *P*_*GAL1*_ *-* CrtB *+ P*_*GAL7/10*_ *-*CrtI		3.28 g/L	not reported	(Shi et al., 2019)
	*P*_*GAL1*_ *-*CrtE *+ P*_*GAL10*_ *-* CrtB *+ P*_*GAL7*_ *-*CrtI		120.36 mg/L	20.63 mg/g DCW	(Watcharawipas et al., 2021)
Gene Copy Number					
A) δ-integration in Ty1 retro-transposon sites	multiple copies of *crtEMB2, crtBM1, crtI* with optimal combination of 2(*crtEMB2*) + *crtBM1 + crtI*		120 mg/L	41.8 mg/g CDW	(Hong et al., 2019)
B) HapAmp System	CrtYB(E83K) + CrtI		not reported	25 mg/g CDW	(Peng et al., 2022)
Summary of Block Strategies for Lycopene Production in *S. cerevisiae*					
Downregulation of Competing Nodes	*ERG9* downregulation	*P*_*HXT1*_ *- ERG9* repressible promoter + *ΔEXG1*	2.1 g/L	not reported	(Liu et al., 2024)
	​	fusion to PEST degron	20.58 mg/L	not reported	(Fan et al., 2024)
Deletion of competing nodes and Repressors	*ΔDPP1* and *ΔLPP1* and downregulation of *ERG9* + *ΔROX1* and *ΔMOT3* (repressors)		120 mg/L	41.8 mg/g CDW	(Hong et al., 2019)
Relief of sterol-mediated feedback regulation	*tHMG1* (truncated)		1.65 g/L	55.5 mg/g DCW	(Chen et al., 2016)
	*tHMG1* (truncated)		not reported	10.1 mg/g DCW	(Xu et al., 2021)

Improving ethanol reassimilation represents another key route for cytosolic acetyl-CoA enrichment. Shi et al. (2019) enhanced this pathway by overexpressing native yeast *ALD6* and *ADH2* alongside a heterologous ACS from *Salmonella enterica*, achieving a lycopene shake-flask titer of 289 mg/L. In a complementary study focusing on downstream lipid structural engineering, Ma et al. (2019) utilized a similar enzymatic configuration to successfully elevate intermediate product accumulation to 10.3 mg/g cdw under aerobic conditions (Table 2).

Consistently, independent evidence from adaptive laboratory evolution (ALE) studies underscores the critical importance of these specific metabolic nodes for driving acetyl-CoA availability (Zhou et al., 2023). Their evolved strain, (yZK006) demonstrated the upregulation of key ethanol-utilization genes (*ADH2*, *ALD4*, and *ACS1*) alongside core β-oxidation components (*FAA2*, *POX1*, and *POT1*), while concurrently downregulating *ADH6* (Zhou et al., 2023). This evolutionary selection pressure directly mirrors the push rationale, demonstrating that optimizing the conversion of endogenous ethanol and fatty acid pools into acetyl-CoA is a highly favored physiological mechanism for maximizing downstream metabolic flux.

Reducing acetyl-CoA consumption has also been successfully employed to preserve intracellular precursor pools. Su et al. (2022) replaced the native promoter of *ACC1*, a key enzyme in fatty acid biosynthesis, with the growth-responsive *PDC1* promoter, thereby reducing acetyl-CoA diversion and doubling lycopene production. Additionally, co-expression of *eutE* and *atoB* using a self-cleaving 2A peptide improved flux through the MVA pathway. When combined with evolved diploid strains such as W2-A-5, this strategy enabled lycopene titers as high as 68 mg/g DCW(Table 2).

Collectively, these findings highlight acetyl-CoA pool optimization as a critical determinant of lycopene biosynthetic capacity. By simultaneously enhancing precursor supply, improving metabolic recycling, and minimizing competing pathways, engineered *S. cerevisiae* strains can achieve substantially improved production performance.

### 4.2 Mevalonate Pathway Engineering

The mevalonate (MVA) pathway constitutes the central metabolic route for isoprenoid biosynthesis in *Saccharomyces cerevisiae* and represents a critical control point for lycopene production (Figure 1). Despite its native presence, flux through this pathway is inherently constrained by tight regulatory mechanisms, feedback inhibition, and competition with essential cellular processes such as sterol biosynthesis. As a result, the availability of the key intermediates isopentenyl pyrophosphate (IPP) and dimethylallyl pyrophosphate (DMAPP) is often insufficient to sustain high-level carotenoid production (Luo et al., 2020; Xia et al., 2024).

To overcome these limitations, extensive metabolic engineering efforts have focused on increasing pathway flux through enzyme deregulation and targeted overexpression. Among these, the use of *tHMG1*, a truncated and deregulated form of HMG-CoA reductase, has emerged as a cornerstone strategy to relieve pathway bottlenecks and enhance carbon flux toward isoprenoid precursors. For example, stepwise overexpression of key MVA pathway genes, including *ERG10*, *ERG12*, *ERG20*, and *tHMG1*, resulted in a cumulative increase in lycopene production, with up to a 1.8-fold improvement compared to the parental strain (Huang et al., 2024). Notably, *ERG20* and *tHMG1* contributed most significantly to this enhancement, whereas overexpression of *ERG13* negatively affected cell growth and overall productivity.

Consistent with these findings, Ma et al. (2019) demonstrated that strengthening flux through the MVA pathway is a prerequisite for achieving high lycopene titers. Their engineered strain, incorporating both pathway enzymes (CrtE, CrtB, CrtI) and upstream flux-enhancing modules, achieved 56.2 mg/g DCW prior to further optimization of storage and transport mechanisms. These results collectively highlight the importance of coordinated pathway amplification rather than single-gene interventions (Table 2).

In addition to enzyme-level modifications, promoter engineering has played a crucial role in fine-tuning pathway activity. Constitutive promoters such as *TEF1* and *PGK1* enable stable expression, whereas inducible systems such as *GAL1/10* and *ADH2* facilitate dynamic control and growth–production phase separation. Particularly, GAL-based systems have enabled high-density fermentations, achieving lycopene titers as high as 3.28 g/L (Shi et al., 2019), demonstrating the importance of temporal regulation in maximizing pathway efficiency (Table 2).

Beyond native pathway optimization, synthetic alternatives have been explored to bypass inherent regulatory constraints. The Isopentenol Utilization Pathway (IUP), a two-step synthetic route that converts isopentenol into IPP and DMAPP, provides an NADPH-independent and ATP-driven alternative to the endogenous MVA pathway. Integration of IUP into engineered yeast strains has, in several cases, resulted in improved precursor supply and enhanced product yields compared to native pathway engineering alone (Luo et al., 2020; Xia et al., 2024).

Overall, the MVA pathway functions as a central metabolic hub that dictates the efficiency of lycopene biosynthesis. Strategies combining enzyme deregulation, pathway amplification, and dynamic expression control provide a powerful framework for overcoming native limitations and achieving industrially relevant production levels in *S. cerevisiae*.

### 4.3 NADPH regeneration and redox balancing

NADPH availability represents a critical determinant of lycopene biosynthesis in *Saccharomyces cerevisiae*, as it provides the reducing power required for multiple steps within the mevalonate (MVA) pathway (Figure 1). In particular, the conversion of HMG-CoA to mevalonate catalyzed by HMG-CoA reductase constitutes a major NADPH-dependent step, making redox balance a key limiting factor for pathway efficiency. Consequently, insufficient NADPH supply can restrict carbon flux even when precursor availability is enhanced, highlighting the importance of coordinated cofactor engineering.

To address this limitation, several metabolic engineering strategies have been developed to enhance intracellular NADPH regeneration. One of the most widely applied approaches involves strengthening the oxidative pentose phosphate pathway (PPP), a primary source of cytosolic NADPH. Overexpression of *ZWF1*, encoding glucose-6-phosphate dehydrogenase, has been shown to significantly increase NADPH generation and improve lycopene production (Zhao et al., 2015). This strategy has been further enhanced by integrating additional pathway modifications, such as the introduction of a phosphoketolase shunt, which simultaneously improves carbon flux toward both acetyl-CoA and NADPH (Fan et al., 2024).

In addition to cytosolic NADPH generation, mitochondrial NADPH metabolism has also been targeted. Overexpression of *POS5*, which encodes a mitochondrial NADH kinase, has been demonstrated to increase NADPH availability and enhance lycopene biosynthesis without compromising cellular growth (Huang et al., 2024; Zhao et al., 2015). These findings highlight the importance of cross-compartmental redox balance in supporting efficient metabolite production.

Regulatory strategies have likewise been explored to globally enhance NADPH-generating pathways. The transcription factor *STB5*, known to activate genes involved in NADPH production, has been co-expressed with membrane engineering targets such as *OLE1*, resulting in substantial increases in lycopene accumulation (Hong et al., 2019). This approach underscores the effectiveness of systems-level regulation in coordinating redox metabolism. However, it is important to note that excessive NADPH supply, in the absence of balanced metabolic demand, may impose a metabolic burden and negatively impact cell growth (Bergman et al., 2019).

Collectively, these strategies demonstrate that redox cofactor engineering is essential for unlocking the full biosynthetic potential of the MVA pathway. By integrating NADPH regeneration with precursor supply optimization and dynamic pathway control, engineered yeast strains can achieve improved flux distribution and enhanced lycopene production, paving the way for industrially viable carotenoid biosynthesis.

## 5 Pull–Optimizing pathway flux and product accumulation

**FIGURE 2:**
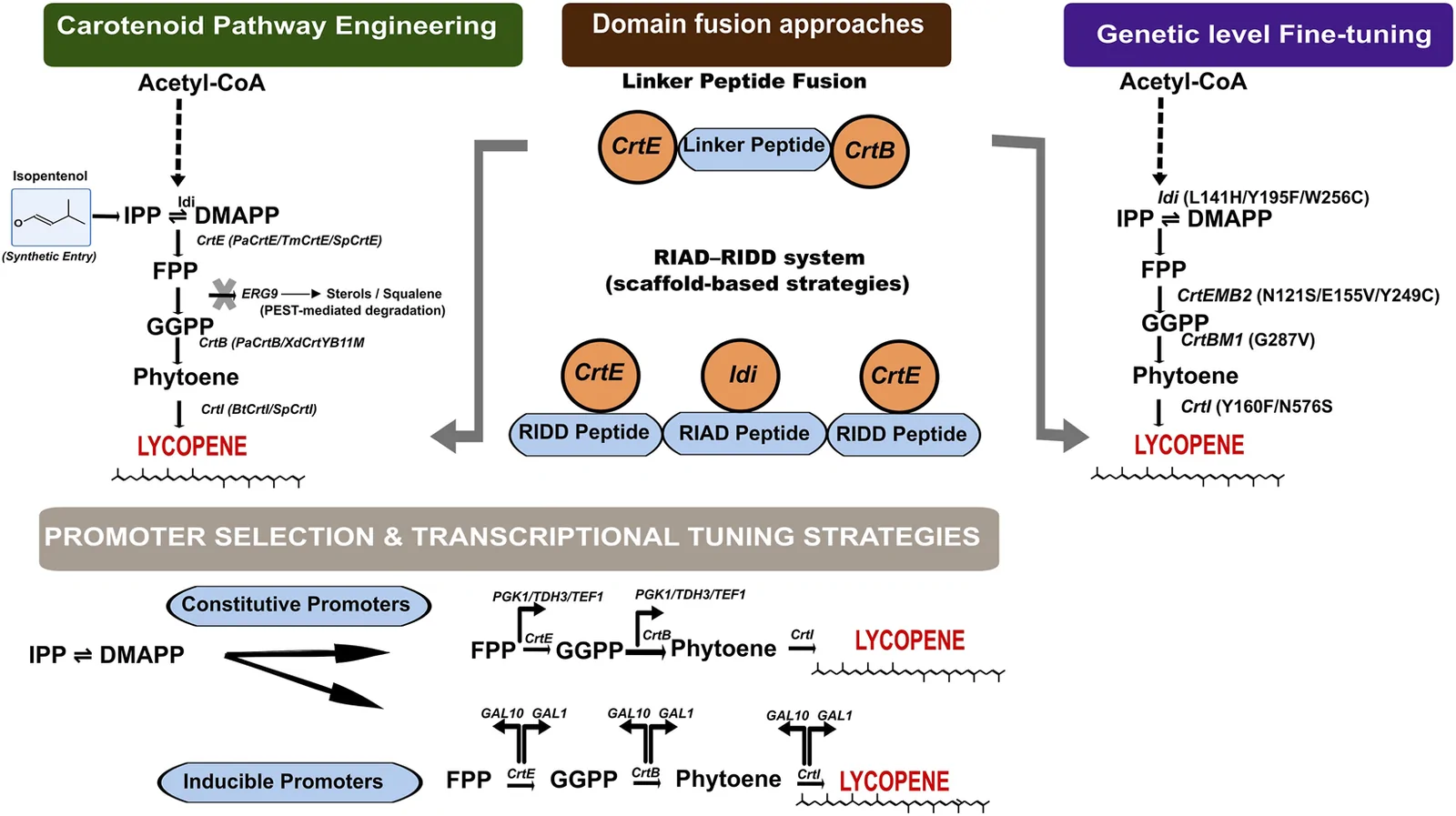
Pull strategy for carotenoid pathway optimization, spatial strategies and transcriptional tuning.Carotenoid Pathway Engineering & Domain Fusion Approaches: Details spatial organization tactics including physical domain fusion of biosynthetic enzymes *via* a flexible linker peptide (CrtE-linker-CrtB) and the non-covalent assembly of multi-enzyme complexes utilizing synthetic RIAD and RIDD peptide anchoring scaffolds (CrtE-RIDD and Idi-RIAD). Genetic-level Fine-tuning & Transcriptional Strategies: Highlights the systematic balancing of key pathway nodes through strategic promoter selection, comparing the utilization of robust constitutive promoters with dynamic, regulated inducible promoters to control expression patterns and eliminate toxic intermediate accumulation during lycopene overproduction.

While push strategies enhance precursor availability and expand the metabolic capacity of the host, efficient conversion of these intermediates into lycopene requires precise optimization of pathway flux, enzyme performance, and gene expression. Therefore, the “pull” strategy in metabolic engineering focuses on strengthening flux toward the target product by improving enzyme efficiency, pathway organization, and product accumulation capacity. In the context of lycopene biosynthesis, this involves optimizing the performance of heterologous carotenoid enzymes, fine-tuning gene expression levels, and facilitating efficient substrate channeling to minimize intermediate loss and metabolic imbalance (Figure 2).

### 5.1 Carotenoid pathway (crt) engineering

One of the central challenges in lycopene biosynthesis is the limited compatibility of heterologous carotenoid enzymes with the yeast cellular environment, particularly for membrane-associated steps. As a result, improving enzyme performance and pathway efficiency has become a key focus of pull strategies. A fundamental approach involves the identification and optimization of *crt* genes from diverse biological sources to construct highly active and compatible biosynthetic modules. Rather than relying on a single organism, studies have demonstrated that combining enzymes from different species can significantly enhance pathway performance. For example, the combination of CrtE from *Taxus x media*, CrtB from *Pantoea agglomerans*, and CrtI from *Blakeslea trispora* has been shown to support efficient lycopene production in yeast (Chen et al., 2016). These findings highlight the importance of modular pathway design and enzyme compatibility.

Beyond gene selection, protein engineering and spatial organization strategies have been employed to further enhance pathway efficiency. Domain fusion approaches, such as CrtB–CrtI–CrtY constructs, can improve catalytic efficiency and reduce intermediate accumulation; however, their performance is context-dependent and may not always outperform modular expression systems (Xu et al., 2021). The RIAD-RIDD system is a scaffolding strategy used in metabolic engineering that utilizes a pair of short, strongly interacting peptide tags to colocalize sequential pathway enzymes into close physical proximity (Eun and Lee, 2024; Kang et al., 2019). Applying this framework, the RIAD–RIDD system enabled the spatial co-localization of sequential pathway nodes by pairing the upstream isomerase Idi (fused with the RIAD tag) with the downstream prenyltransferase CrtE (fused with the RIDD tag). This targeted spatial organization significantly enhanced substrate channeling, minimizing the diffusion of pathway intermediates and driving lycopene titers to an industrially relevant 2.3 g/L in fed-batch fermentations (Kang et al., 2019) (Table 2).

Fine-tuning pathway architecture at the genetic level has also proven essential. Optimization of gene copy number, promoter strength, and genomic integration sites allows for precise control of metabolic flux. Moderate increases in *crt* gene copy number enhance production, whereas excessive expression can impose metabolic burden and reduce cell fitness (Liu et al., 2024). In parallel, targeted protein engineering, including point mutations (Chen et al., 2018; Jing et al., 2021) that improve enzyme stability and catalytic efficiency, has been shown to significantly enhance lycopene titers (Xie et al., 2015; Zhou et al., 2023).

Pull engineering does not operate in isolation: the efficiency with which the carotenoid module draws carbon from the FPP node also depends on the suppression of competing sinks at that node, which is addressed as a block strategy in Section 6.1. Collectively, these strategies demonstrate that effective pull engineering relies on a combination of enzyme optimization, pathway organization, and expression control, enabling efficient conversion of precursors into the desired product.

### 5.2 Promoter and gene copy tuning

Fine-tuning transcriptional strength and gene dosage represents a critical pull strategy for optimizing metabolic flux toward lycopene production in *Saccharomyces cerevisiae*. Unlike precursor supply, which determines upstream capacity, precise control of gene expression directly governs pathway balance, intermediate accumulation, and overall productivity.

Constitutive promoters, particularly strong glycolytic promoters such as PTEF1, *TDH3* and *PGK1*, are widely employed to achieve stable and high-level expression of carotenoid biosynthetic genes. Several studies have demonstrated that constitutive expression of *crtE*, *crtB*, and *crtI* under strong promoters can support efficient lycopene production, often outperforming inducible systems under standard batch conditions (Li et al., 2019; Shi et al., 2019; Sun et al., 2016; Xu et al., 2021). This strategy ensures continuous flux through the pathway but may impose metabolic burden if not properly balanced.

In contrast, inducible promoter systems provide dynamic control over pathway activation by decoupling biomass formation from product synthesis. GAL-regulated promoters (*GAL1*, *GAL7*, *GAL10*) are among the most extensively used systems, enabling low expression during exponential growth and strong induction in later phases. This temporal regulation is particularly advantageous in lycopene production, where product accumulation can exert cytotoxic effects. For instance, dynamic induction strategies have been shown to improve carotenoid yields and maintain cellular fitness, while also demonstrating scalability in bioreactor systems (Shi et al., 2019; Watcharawipas et al., 2021).

Beyond promoter selection, gene copy number plays a decisive role in controlling metabolic flux. While multicopy plasmids such as 2μ vectors enable rapid amplification, they often suffer from genetic instability and increased metabolic burden. As a result, chromosomal integration strategies have gained prominence. Techniques such as δ-integration targeting Ty1 retrotransposon sites allow stable multicopy insertion, with lycopene titers varying depending on genomic locus and copy number (Hong et al., 2019). More recently, *in vivo* amplification approaches, including antibiotic-driven selection systems and adaptive amplification strategies such as HapAmp, have enabled further expansion of gene dosage, in some cases reaching dozens of pathway copies per cell (Peng et al., 2022; Yang and Zhang, 2018).

However, increasing gene copy number does not linearly translate into improved production. Excessive expression can lead to metabolic imbalance, accumulation of toxic intermediates, and reduced cell growth (Liu et al., 2024; Xie et al., 2015; Zhou et al., 2023). Therefore, optimal pathway performance requires balanced expression across the biosynthetic cascade. For example, high expression of upstream genes such as *tHMG1* can enhance precursor supply, whereas moderated expression of downstream enzymes (*crtYB*, *crtI*) helps prevent metabolic overload and improves overall yield.

In summary, promoter engineering and gene copy tuning serve as powerful regulatory levers for controlling pathway flux in lycopene biosynthesis. The integration of dynamic expression systems, stable genomic amplification strategies, synthetic pathway integration, and pathway balancing principles enables precise control over metabolic networks, ultimately facilitating higher efficiency and scalability in engineered yeast platforms.

## 6 Block–Redirecting flux by suppressing competing pathways

**FIGURE 3:**
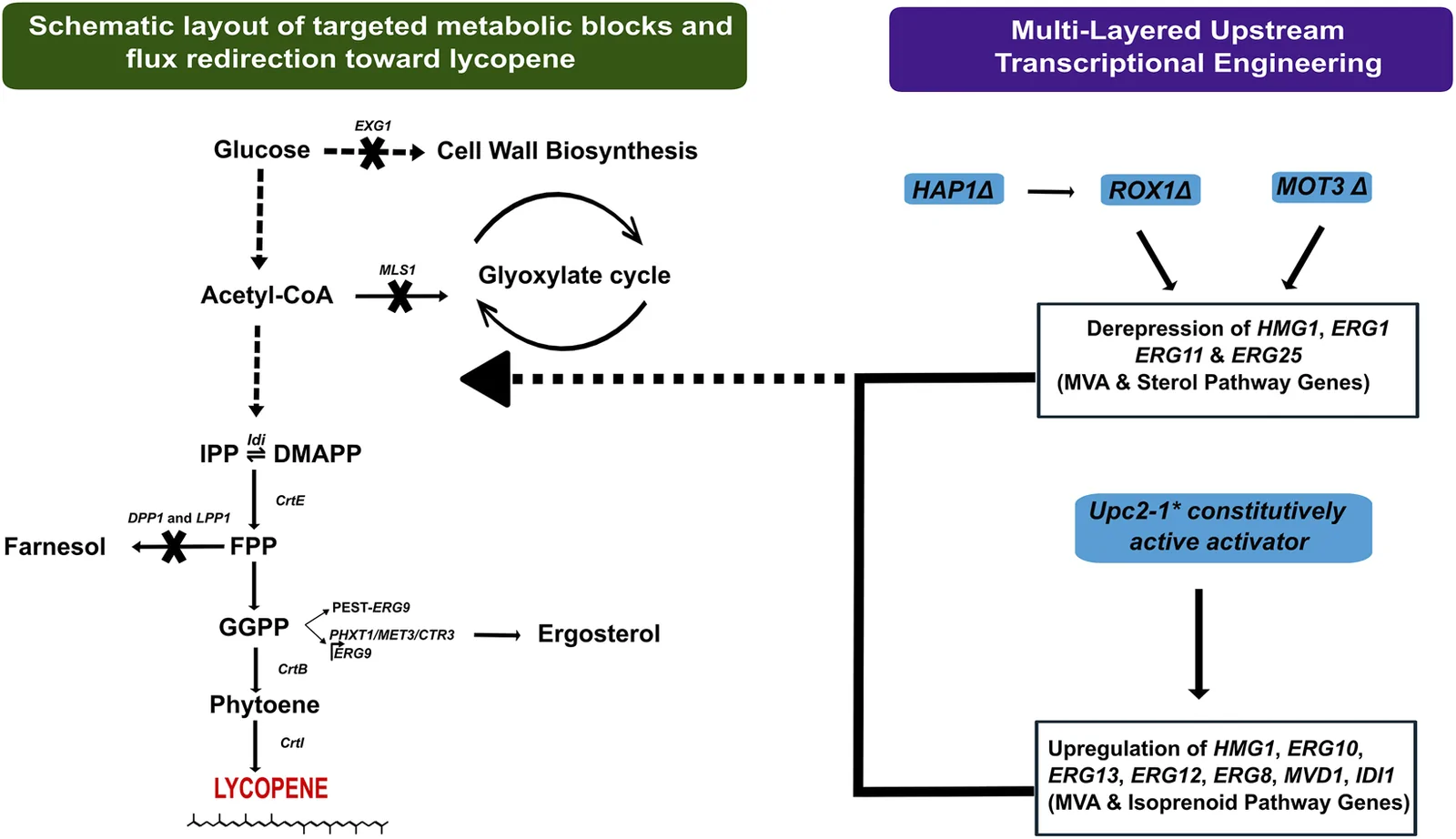
Metabolic block and upstream transcriptional engineering strategies. Targeted metabolic blocks and flux redirection: Highlights specific genetic deletions (X) introduced to stop competitive carbon drainage into side pathways. These include the abolishment of *EXG1* to suppress cell wall biosynthesis, *MLS1* to disrupt the glyoxylate cycle, DPP1 and LPP1 to prevent farnesol accumulation from the farnesyl diphosphate (FPP) node and regulated downregulation strategies for *ERG9* to minimize flux leak into ergosterol formation. Multi-layered upstream transcriptional engineering: Outlines the deletion of transcriptional repressors (*HAP1*Δ, *ROX1*Δ, and *MOT3*Δ) to achieve the passive derepression of key MVA and sterol pathway genes (*HMG1*, *ERG1*, *ERG11*, *ERG25*). This layer operates in tandem with the expression of the constitutively active transcriptional activator Upc2-1*, driving the strong upregulation of the core upstream mevalonate and isoprenoid pathway genes (*HMG1*, *ERG10*, *ERG13*, *ERG12*, *ERG8*, *MVD1*, *IDI1*) to systematically push precursor flux toward lycopene.

Despite improvements in precursor supply and pathway flux, a significant portion of carbon is still diverted into competing endogenous pathways, particularly sterol biosynthesis. This metabolic competition represents a major bottleneck, necessitating targeted interventions to suppress competing nodes and relieve regulatory constraints that limit carbon flux toward lycopene production (Figure 3).

### 6.1 Downregulation of competing nodes

Redirecting carbon flux toward lycopene biosynthesis in *Saccharomyces cerevisiae* requires the selective suppression of competing metabolic pathways that consume key intermediates. Among these, sterol biosynthesis represents the most significant competing route, as it diverts farnesyl pyrophosphate (FPP) away from carotenoid production (Figure 3).

A critical control point in this competition is *ERG9*, which encodes squalene synthase and catalyzes the first committed step toward ergosterol biosynthesis. Since *ERG9* is essential for cell viability, complete gene deletion is not feasible. Instead, tunable downregulation strategies have been widely employed to reduce flux through this branch. These include the use of condition-responsive promoters such as *PHXT1*, *MET3*, and *CTR3*, as well as ergosterol-sensitive regulatory systems that enable dynamic control of *ERG9* expression (Huang et al., 2024; Liu et al., 2024; Xie et al., 2015).

Beyond transcriptional regulation, post-translational strategies have also been explored. Targeted protein degradation *via* fusion of a PEST degron to *ERG9* provides a means to reduce enzyme activity without genetic knockout. This approach has been shown to significantly enhance carotenoid production while maintaining cellular fitness, achieving improvements comparable to promoter engineering strategies (Fan et al., 2024).

The targeted downregulation of *ERG9*—coupled with the simultaneous deletion of DPP1 and LPP1—which catalyze the conversion of farnesyl pyrophosphate (FPP) to farnesol, pulling flux away from lycopene synthesis, successfully blocked alternative pathways to farnesol and squalene. This coordinated metabolic diversion redirected precursor flux entirely toward the heterologous carotenoid pathway, yielding robust cell growth and a substantial increase in lycopene production (Hong et al., 2019).

Transcriptomic analyses further support the importance of suppressing competing pathways. During high-level carotenoid production, coordinated downregulation of sterol biosynthesis genes (*ERG1, ERG11, ERG25, ERG26, ERG6, ERG3,* and *ERG4*) has been observed, suggesting an intrinsic regulatory shift that favors lycopene accumulation (Zhou et al., 2023).

In addition to sterol pathway modulation, other competing metabolic routes have been targeted to improve precursor availability. For example, deletion of *MLS1* increases intracellular acetyl-CoA levels by reducing glyoxylate cycle flux, thereby enhancing precursor supply for the MVA pathway (Krivoruchko et al., 2013). Similarly, disruption of *EXG1*, which is involved in cell wall biosynthesis, redirects glucose-derived carbon toward central metabolism and lycopene production, improving both growth and product titers (Liu et al., 2024).

Overall, these strategies demonstrate that selective weakening of competing metabolic nodes—while preserving essential cellular functions—is a key principle for efficient flux redirection. By minimizing carbon loss to alternative pathways, engineered yeast strains can achieve more effective utilization of precursors for lycopene biosynthesis.

### 6.2 Relief of feedback inhibition and transcriptional repression

Lycopene biosynthesis in *Saccharomyces cerevisiae* is tightly constrained by multi-layered regulatory mechanisms, including feedback inhibition and transcriptional repression within the mevalonate (MVA) and sterol biosynthesis pathways (Figure 3). These regulatory controls act as intrinsic metabolic brakes, limiting flux even when precursor availability and pathway capacity are enhanced. Consequently, relieving these constraints is essential for achieving high-level carotenoid production.

A central target in this context is HMG-CoA reductase (HMGR), the rate-limiting enzyme of the MVA pathway. Native HMGR is subject to sterol-mediated feedback regulation and degradation. Truncation of its N-terminal regulatory domain (*tHMG1*) effectively bypasses this control, enabling sustained enzyme stability and increased flux through the pathway. This modification has consistently been shown to significantly enhance isoprenoid and carotenoid production across multiple studies (Chen et al., 2016; Scalcinati et al., 2012; Xu et al., 2021).

Transcriptional repression further restricts pathway activity. Repressors such as *ROX1* and *MOT3* downregulate sterol- and hypoxia-responsive genes under aerobic conditions, thereby limiting isoprenoid biosynthesis. Deletion of repressors such as *ROX1* and *MOT3*, particularly *ROX1*, leads to derepression of key MVA and sterol pathway genes (*HMG1*, *ERG1*, *ERG11*, *ERG25*, among others). To redirect the increased flow away from the sterol pathway, the deletion of *ROX1* and *MOT3* combined with the downregulation of *ERG9* resulted in substantial increases in lycopene production (Hong et al., 2019).

Beyond repressor deletion, transcriptional activator engineering offers an alternative route to maximize metabolic throughput. Overexpression of the constitutively active transcription factor mutant Upc2-1 upregulates several key genes in isoprenoid biosynthesis, improving upstream precursor pools (Chai et al., 2024; Li et al., 2025). Similarly, deletion of *HAP1*, a regulator of heme-dependent transcription, indirectly alleviates repression by reducing *ROX1* expression, effectively mimicking the rox1Δ phenotype and promoting increased flux through the MVA pathway (Gao et al., 2022). Integrating these upstream pull-and-push mechanisms with a downregulated *ERG9* node has proved pivotal in steering metabolic flux away from sterols to optimize terpenoid synthesis (Chai et al., 2024; Li et al., 2025).

In addition to transcriptional regulation, feedback inhibition can also be mitigated by disrupting downstream metabolic sinks. Downregulating *ERG9* chokes off the competitive sterol pathway, depleting downstream sterol pools and alleviating negative feedback on upstream rate-limiting steps. This restriction minimizes carbon drain while triggering a compensatory transcriptional upregulation of the core mevalonate (MVA) pathway, cooperatively maximizing flux toward the production of lycopene and other valuable terpenoids (Fan et al., 2024; Huang et al., 2024; Paddon et al., 2013; Xie et al., 2015).

Collectively, these strategies demonstrate that relieving regulatory constraints—through enzyme stabilization, transcriptional rewiring, and feedback control—is essential for unlocking the full biosynthetic potential of the MVA pathway. When integrated with precursor supply enhancement and flux optimization strategies, these interventions enable efficient and scalable lycopene production in engineered yeast systems.

## 7 Integrated strategies for optimizing lycopene production in *Saccharomyces cerevisiae*

The push–pull–block strategies discussed above define the core logic of flux redirection toward lycopene, yet rational intervention at the pathway level alone is rarely sufficient to reach industrially relevant performance. As flux is intensified, new constraints emerge that are not pathway-encoded but cellular and process-level in nature: the hydrophobic product must be stored or exported, the host must tolerate the oxidative and membrane stress that accompanies high-level accumulation, and biosynthesis must be sustained under fermentation conditions that differ substantially from laboratory cultivation. The strategies described in this section address these constraints. Rather than constituting a fourth category alongside push, pull, and block, they form an enabling layer that determines how much of the engineered flux can actually be realized: secretion and storage engineering (Section 7.1) and stress mitigation (Section 7.2) extend the capacity of the cell to sustain a strong pull; adaptive evolution, random mutagenesis (Section 7.3), and genome restructuring (Section 7.4) act across all three components simultaneously by uncovering non-intuitive optima that rational design cannot predict; fermentation optimization (Section 7.5) and carbon source selection (Section 7.6) shape precursor and cofactor supply at the process level, thereby reinforcing push; and multi-omics- and machine learning-guided design (Section 7.7) provides the system-level insight required to coordinate these interventions. Together, these approaches mark the transition from pathway-centric engineering toward whole-cell and whole-process optimization.

### 7.1 Enhancing lycopene secretion and storage

The highly hydrophobic nature of lycopene imposes significant limitations on its accumulation in *Saccharomyces cerevisiae*, often leading to intracellular aggregation, membrane stress, and reduced cellular fitness. As pathway flux is intensified through push–pull–block strategies, product accumulation itself becomes a new bottleneck. Therefore, engineering cellular systems to improve lycopene storage and secretion has emerged as a critical post-pathway optimization strategy.

One major approach involves transporter engineering to facilitate active export of hydrophobic carotenoids. ATP-binding cassette (ABC) transporters have been extensively explored for this purpose. Overexpression of the endogenous transporter Snq2p significantly enhanced carotenoid secretion, resulting in approximately fourfold increases in extracellular β-carotene levels and moderate improvements in intracellular accumulation. When combined with increased ATP supply and membrane engineering *via OLE1* overexpression, secretion efficiency was further amplified (Bu et al., 2020). Although other transporters such as Pdr11p have been evaluated, Snq2p remains one of the most effective candidates for carotenoid efflux.

In parallel, enhancing intracellular storage capacity through lipid droplet (LD) engineering has proven highly effective. A substantial proportion of carotenoids accumulates within lipid droplets rather than cellular membranes, making LDs critical storage organelles. Increasing LD formation through *OLE1* overexpression and *FLD1* deletion has been shown to improve lycopene accumulation and cellular tolerance (Ma et al., 2019). Additionally, supplementation with unsaturated fatty acids such as oleic and palmitoleic acid further enhances neutral lipid content and carotenoid storage capacity, significantly boosting production levels (Sun et al., 2016).

Complementing these endogenous strategies, heterologous lipid-binding systems have been introduced to further improve carotenoid sequestration and transport. Expression of human saposin B (hSapB), a lipid-binding protein, enabled efficient intracellular encapsulation of hydrophobic compounds, resulting in substantial increases in carotenoid accumulation. Moreover, fusion of hSapB with secretion signals facilitated extracellular release of compounds such as β-carotene and squalene, demonstrating its dual functionality in storage and secretion enhancement (Fan et al., 2024).

Collectively, these strategies highlight that effective lycopene production requires not only pathway optimization but also adaptation of the cellular environment to accommodate hydrophobic products. By integrating transporter engineering, lipid droplet expansion, and synthetic sequestration systems, *S. cerevisiae* can be transformed into a product-tolerant and high-capacity production platform capable of achieving industrially relevant carotenoid titers.

### 7.2 Stress mitigation and growth-decoupled production in lycopene-producing *S. cerevisiae*

High-level lycopene production imposes a substantial metabolic burden on *Saccharomyces cerevisiae*, leading to oxidative stress, membrane destabilization, protein folding stress, and impaired cellular growth. As pathway flux is intensified through push–pull–block strategies, maintaining cellular robustness becomes a critical determinant of production performance. Therefore, modern metabolic engineering approaches increasingly incorporate host-level interventions to enhance stress tolerance and decouple metabolite production from growth.

While NADPH availability has been discussed as a key driver of metabolic flux in the push layer, its role here is considered from a different perspective, as a critical factor in maintaining redox balance and mitigating oxidative stress under high-level lycopene production.

Redox imbalance represents one of the primary stress factors during lycopene biosynthesis, largely due to increased NADPH demand. Enhancing NADPH regeneration through transcriptional and enzymatic strategies has been shown to alleviate oxidative stress and improve productivity. Overexpression of the transcription factor *STB5*, which regulates pentose phosphate pathway (PPP) genes such as *ZWF1*, *GND1*, and *GND2*, enhances NADPH supply and supports higher lycopene titers (Hong et al., 2019). Similarly, direct overexpression of *ZWF1* and *POS5* further strengthens intracellular reducing power and improves carotenoid production (Huang et al., 2024; Zhao et al., 2015).

In parallel, membrane stress caused by the accumulation of hydrophobic carotenoids can be mitigated through lipid remodeling. Overexpression of *OLE1*, encoding a Δ9-fatty acid desaturase, increases the proportion of unsaturated fatty acids, thereby improving membrane fluidity and tolerance to lycopene accumulation. Co-expression of *OLE1* with *STB5* has been shown to synergistically enhance production levels. Additionally, activation of phospholipid biosynthesis through *INO2* overexpression, as well as supplementation with unsaturated fatty acids such as oleic acid, further improves membrane dynamics and cellular resilience (Chen et al., 2016; Ma et al., 2019; Sun et al., 2016).

Protein folding stress represents another important limitation, particularly under high expression of heterologous enzymes. Accumulation of misfolded proteins can trigger the unfolded protein response (UPR), negatively impacting cell viability and productivity. To address this, dynamic expression strategies have been employed to delay pathway activation until after biomass accumulation. For example, growth-phase-responsive promoters such as *PHXT1* enable temporal separation of growth and production, thereby reducing cellular stress and improving overall yields (Xie et al., 2015).

Beyond genetic strategies, regulatory circuits that respond to intracellular stress signals have also been explored. Overexpression of *RIM15*, which activates antioxidant defense pathways *via* Yap1, enhances tolerance to reactive oxygen species (ROS) and stabilizes redox homeostasis during high-level production (Li et al., 2025).

At the process level, growth–production decoupling is further reinforced through two-stage fermentation strategies. In this approach, cells are first cultivated under optimal growth conditions, followed by induction of lycopene biosynthesis in a second phase. This separation reduces metabolic burden during biomass formation and enables higher final product titers (Bu et al., 2020). Integration of such process-level strategies with genetic robustness has enabled substantial improvements in industrial-scale production.

Collectively, these findings demonstrate that successful lycopene production requires not only pathway optimization but also the stabilization of the cellular environment under production stress. By integrating redox balancing, membrane remodeling, dynamic expression control, and process engineering, *S. cerevisiae* can be transformed into a robust and stress-tolerant production platform capable of sustaining high-level carotenoid biosynthesis.

### 7.3 Adaptive evolution and random mutagenesis

To further enhance lycopene production in *Saccharomyces cerevisiae*, recent strategies have expanded beyond rational metabolic engineering toward evolutionary approaches that leverage the organism’s intrinsic adaptability. Adaptive laboratory evolution (ALE), random mutagenesis, and genome-scale restructuring have emerged as powerful complementary tools for overcoming hidden metabolic bottlenecks and improving strain robustness.

One of the most effective applications of ALE in lycopene-producing strains involves adaptation to oxidative stress. Given that lycopene itself functions as an antioxidant, applying selective pressures such as hydrogen peroxide (H_2_O_2_) enables the enrichment of strains capable of both enhanced stress tolerance and increased carotenoid production. For instance, ALE under oxidative conditions has yielded evolved strains with up to threefold higher lycopene content compared to the parental strain, accompanied by upregulation of genes involved in lipid metabolism and isoprenoid biosynthesis (Reyes et al., 2014). More recently, this strategy has been integrated with upstream pathway optimization, resulting in strains capable of producing up to 8.15 g/L lycopene under fed-batch conditions—representing one of the highest reported titers in yeast (Zhou et al., 2023).

Complementing ALE, random mutagenesis was used in the same study to generate genetic diversity and accelerate strain improvement (Zhou et al., 2023) and utilized atmospheric and room-temperature plasma (ARTP) mutagenesis due to its high efficiency and controllability. In combination with oxidative evolution, ARTP resulted in high-performing mutants with enhanced stress resistance and metabolic capacity.

### 7.4 Genome restructuring

Large-scale genome restructuring offers an additional dimension for strain optimization. Alterations in ploidy, such as transitioning from haploid to diploid states, have been shown to improve tolerance to metabolic burden and enhance production stability (Xie et al., 2015). At a more advanced level, the SCRaMbLE system—developed within the Synthetic Yeast 2.0 framework—enables rapid combinatorial genome rearrangement. Application of SCRaMbLE to lycopene-producing strains has resulted in dramatic improvements in production, with reports of over 100-fold increases in carotenoid titers through iterative cycles of genomic rearrangement and selection (Zhang et al., 2021).

Collectively, these evolutionary strategies highlight the limitations of purely rational design approaches in complex metabolic systems. By introducing genetic diversity and applying targeted selection pressures, evolutionary engineering enables the discovery of advantageous phenotypes that may not be predictable through conventional pathway optimization. Integration of these approaches with push–pull–block strategies provides a powerful framework for developing robust, high-yielding *S. cerevisiae* strains suitable for industrial-scale lycopene production.

### 7.5 Fermentation process optimization

While metabolic engineering establishes the genetic framework for lycopene biosynthesis, fermentation process optimization plays a crucial role in translating this potential into industrial-scale production. Process-level interventions influence cellular physiology, metabolic flux distribution, and product stability, thereby serving as a critical layer for maximizing yield and productivity.

One widely explored strategy involves the use of metabolic inhibitors to prevent the conversion of lycopene into downstream carotenoids. In native carotenogenic systems such as *Blakeslea trispora*, inhibitors of lycopene cyclase—such as imidazole and piperidine—have been shown to significantly enhance lycopene accumulation by blocking its conversion to β-carotene (Choudhari et al., 2008; Li et al., 2020). While these approaches are less directly applicable to engineered *S. cerevisiae* strains, they highlight the importance of controlling pathway endpoints at the process level.

Media engineering and chemical supplementation also represent key tools for improving lycopene production. The use of oxygen vectors such as n-hexane and n-dodecane enhances oxygen transfer and has been shown to increase lycopene titers by more than twofold. Similarly, supplementation with plant-derived oils rich in unsaturated fatty acids, particularly linoleic acid, improves membrane fluidity and promotes intracellular storage of hydrophobic carotenoids. For example, soybean oil supplementation has been reported to increase lycopene production by over 80% (Li et al., 2020).

Unsaturated fatty acids (UFAs), including linolenic and palmitoleic acid, further contribute to enhanced production by improving membrane dynamics and facilitating carotenoid accumulation. In engineered strains, exogenous UFA supplementation has been shown to significantly increase lycopene titers, likely by both enhancing storage capacity and redirecting acetyl-CoA flux toward the mevalonate pathway (Xu et al., 2021).

Fermentation conditions such as carbon source concentration, pH, dissolved oxygen, and temperature also exert strong effects on production efficiency. Lower glucose concentrations have been shown to improve metabolic balance, while controlled oxygen supply supports both growth and biosynthesis. Notably, reduced fermentation temperatures can enhance membrane unsaturation and fluidity, thereby improving cellular tolerance and increasing lycopene production (Zhang et al., 2021).

At the bioprocess level, growth–production decoupling strategies, such as two-stage fed-batch fermentation, have been widely applied. In this approach, cells are first cultivated under optimal growth conditions, followed by induction of lycopene biosynthesis under controlled environmental parameters. This strategy minimizes metabolic burden during biomass formation and enables higher final titers. Integration of such process-level controls with genetically optimized strains is essential for achieving industrially relevant production.

Overall, fermentation process optimization complements genetic engineering strategies by providing a dynamic and scalable framework for enhancing lycopene production. By fine-tuning environmental conditions, nutrient supply, and process parameters, microbial cell factories can be further optimized to achieve high efficiency and robustness under industrial conditions.

### 7.6 Role of carbon source on precursor and cofactor balancing

Glucose serves as the conventional carbon source to support rapid cell growth and biomass accumulation during the initial growth phase. Typically, glucose feeding is maintained until the culture approaches the stationary phase, at which point it is discontinued and substituted with a secondary carbon source—most notably ethanol—to fuel the energetically demanding lycopene biosynthetic phase (Chen et al., 2016; Shi et al., 2019).

Ethanol is usually utilized as a primary carbon source during the secondary “product accumulation” stage of two-stage fermentations (Ma et al., 2019; Shi et al., 2019). Within the cell, ethanol is oxidized to acetaldehyde and then to acetate, feeding directly into the cytosolic acetyl-CoA pool to drive the mevalonate (MVA) pathway and has been shown to significantly increase β-carotene yields by 73% compared to glucose yields (Reyes et al., 2014).

Glycerol has also been utilized in both *E. coli* and *S. cerevisiae* systems as a highly effective auxiliary carbon source to improve lycopene production (Xie et al., 2015). In yeast fed-batch systems, co-feeding glycerol alongside glucose has been demonstrated to substantially enhance lycopene accumulation while concurrently reducing the accumulation of the competing byproduct squalene (Xie et al., 2015).

Sucrose acts as a low-cost alternative carbon source that is significantly cheaper than both glucose and galactose. In yeast strains where the *GAL80* repressor is deleted, the utilization of sucrose completely relieves the requirement for expensive galactose induction. This substrate strategy has been shown to result in up to a 6-fold increase in heterologous lycopene titers compared to conventional glucose cultivations, without causing any associated growth defects (Watcharawipas et al., 2021).

Citric acid has also been used as a highly efficient carbon source, yielding a 2689-fold increase in lycopene titers to achieve 115.64 mg/L when comparing the final optimized fed-batch process to the initial baseline strain (Li et al., 2019). Citric acid contributes to the formation of the lycopene carbon skeleton and supplies acetyl-CoA to the MVA pathway. This feeding strategy provides a distinct economic advantage because its resulting lycopene yield is double that of glucose, heavily outweighing its raw material cost which is only 1.5 times higher (Li et al., 2019).

Acetate has been used as an exogenous carbon source to simultaneously improve precursor supply and drive pathway regulation. Replacing the native *ERG9* promoter (encoding squalene synthase) with an acetate-repressible promoter PGRE1 promoter dynamically downregulates the competing ergosterol pathway upon acetate addition. This strategy not only enhances the intracellular supply of acetyl-CoA but also concurrently diminishes carbon loss through the squalene sink, successfully redirecting farnesyl pyrophosphate (FPP) flux toward carotenoid synthesis. Consequently, this acetate signaling-based downregulation system resulted in a 42.3% increase in lycopene production (Huang et al., 2024).

Isoprenol supplementation represents a further substrate-level intervention, introduced in the *Mevalonate Pathway Engineering* section as a push strategy because it acts on the upstream supply of isoprenoid precursors rather than on their downstream conversion to lycopene. Exogenous isoprenol bypasses the lengthy and tightly regulated native route from glucose to IPP by feeding a synthetic, heterologous two-step Isopentenol Utilization Pathway (IUP). Beyond shortening the pathway, the IUP is also energetically favourable: it consumes only 2 ATP per IPP, whereas the native MVA and MEP routes require 2 NADPH + 3 ATP and 3 NADPH + 2 ATP per IPP, respectively. Expression of the IUP in the oleaginous yeast *Yarrowia lipolytica* increased the IPP/DMAPP pool 15.7-fold and yielded lycopene titers of 4.2 g/L (Luo et al., 2020). Although demonstrated in *Y. lipolytica*, this strategy illustrates how carbon source selection and precursor-supply engineering converge, and it is directly transferable to *S. cerevisiae*.

Xylose serves as a low-cost, abundant carbon source derived from agricultural lignocellulosic waste. Utilizing xylose in a fed-batch bioreactor yielded 772.8 mg/L of β-carotene, with phytoene and lycopene accumulating as active intermediates. Because xylose metabolism evades glucose-mediated catabolite repression, it transitions the host into a highly respiratory metabolic state that expands the pool of cytosolic acetyl-CoA while simultaneously upregulating the transcription of native genes that are typically rate-limiting—specifically *ACS1* and *HMG1*. While the engineered strain experiences an intracellular NADH/NAD+ redox imbalance that drives glycerol and acetate accumulation, this native transcriptional response effectively eliminates the traditional metabolic engineering requirement to overexpress a feedback-resistant, truncated *HMG1* (*tHMG1*) to maximize downstream terpenoid flux (Sun et al., 2020).

Fatty acids and lipids (including palmitic, linolenic, oleic, and palmitoleic acids) alleviate cell toxicity and membrane stress caused by the high-level accumulation of lipophilic lycopene.

### 7.7 Multi-omics and machine learning-guided design

Recent advances in metabolic engineering have shifted lycopene production in *Saccharomyces cerevisiae* from traditional gene-by-gene optimization toward integrated, data-driven strain design. Multi-omics approaches—including transcriptomics, proteomics, metabolomics, and fluxomics—have played a central role in this transition by enabling system-level characterization of metabolic bottlenecks. These analyses have revealed key constraints related to acetyl-CoA availability, redox and energy balance, stress responses, and competition with sterol biosynthesis, thereby guiding the rational identification of engineering targets (Dagariya et al., 2025; Huang et al., 2026).

Importantly, insights derived from multi-omics data have facilitated modular engineering strategies, allowing coordinated optimization of precursor supply, pathway flux, heterologous enzyme performance, and competing endogenous pathways. This integrative perspective aligns closely with the push–pull–block framework, enabling more precise and systematic intervention across multiple layers of cellular metabolism (Huang et al., 2026).

Beyond descriptive system analysis, machine learning (ML)–assisted approaches have emerged as powerful tools to address the combinatorial complexity of multi-gene pathway optimization. For example, ML-guided gene copy number tuning using non-repetitive coding sequence variants has enabled the prediction of optimal expression configurations, improving both genetic stability and lycopene production in engineered yeast strains (Du et al., 2024). More broadly, ML-integrated frameworks that combine high-throughput data generation with computational modeling have been proposed to support predictive target selection, pathway balancing, and strain optimization (Chai et al., 2025).

Despite these advances, current applications remain largely iterative and semi-predictive, rather than fully autonomous. The development of closed-loop design–build–test–learn (DBTL) cycles—integrating multi-omics data acquisition, ML-driven prediction, and automated strain construction—represents a critical next step for the field. Such systems have the potential to transform metabolic engineering from an empirical discipline into a predictive science.

## 8 Conclusion and future perspectives

Lycopene biosynthesis in *Saccharomyces cerevisiae* has evolved from simple pathway reconstruction toward a highly integrated and multi-layered engineering challenge. Early efforts focused primarily on introducing heterologous carotenoid pathways and enhancing precursor supply; however, it is now evident that achieving industrially relevant production requires coordinated optimization across metabolic, regulatory, cellular, and process levels.

In this review, we have organized existing metabolic engineering strategies within the push–pull–block framework, providing a unified perspective on lycopene production in yeast. “Push” strategies enhance precursor and cofactor availability, particularly acetyl-CoA and NADPH, thereby establishing the metabolic capacity required for isoprenoid biosynthesis. “Pull” strategies improve pathway efficiency through enzyme engineering, pathway organization, and precise control of gene expression, enabling effective conversion of intermediates into lycopene. “Block” strategies, in turn, minimize carbon loss and relieve regulatory constraints by suppressing competing pathways and overcoming feedback inhibition.

Importantly, this review article highlights that these strategies are not independent but highly interconnected. Effective strain design requires simultaneous balancing of precursor supply, pathway flux, and regulatory control. Beyond these core interventions, additional layers of optimization—including storage and secretion engineering, stress mitigation, growth–production decoupling, and adaptive evolution—play essential roles in stabilizing high-flux systems and enabling sustained production. These approaches collectively reflect a shift from pathway-centric engineering toward whole-cell optimization.

Furthermore, recent advances in multi-omics technologies and machine learning have introduced a new paradigm in metabolic engineering. By enabling system-level data integration and predictive design, these approaches offer the potential to move beyond iterative trial-and-error toward rational and automated strain development. However, current implementations remain largely semi-predictive, and the realization of fully integrated design–build–test–learn (DBTL) pipelines represents a key challenge for the field.

Despite significant progress, several limitations remain. Balancing metabolic flux without imposing excessive cellular burden, maintaining genetic stability under industrial conditions, and achieving cost-effective large-scale production continue to be major hurdles. Addressing these challenges will require tighter integration between genetic engineering, systems biology, and bioprocess optimization.

Looking forward, future developments in lycopene biosynthesis are expected to focus on the convergence of dynamic regulation, evolutionary engineering, and data-driven design. The integration of real-time sensing, adaptive control systems, and machine learning-guided optimization holds particular promise for creating robust and self-optimizing microbial cell factories.

Overall, the push–pull–block framework provides not only a conceptual tool for organizing existing knowledge but also a practical design principle for future strain engineering. By adopting this integrated perspective, *S. cerevisiae* can be further developed into a highly efficient and scalable platform for sustainable lycopene production, with broader implications for the biosynthesis of complex terpenoids and other high-value metabolites.
